# Supplementary material for: Flood exposure and pregnancy loss in 33 developing countries
Source: Nat Commun. 2024 Jan 2;15:20. doi: 10.1038/s41467-023-44508-0 (PMC10761804; doi:10.1038/s41467-023-44508-0)
Supplement: Supplementary file 1 — Supplementary Information [file 41467_2023_44508_MOESM1_ESM.pdf]

Supplementary material for

Flood exposure and pregnancy loss in 33 developing countries

Contents

1. **Robustness of main results**

    1.1 **Robustness to the alternative source of flood exposure data**

    1.2 **Robustness to different controls**

    1.3 **Robustness to leaving each region out individually**

    1.4 **Robustness to potential migration**

2. **Effects of different types of floods on pregnancy loss**
3. **Extra data**

    3.1 **Example illustrating our case-control design**

    3.2 **Map of data selection**

    3.3 **DHS Surveys used in the main analysis**

    3.4 **Statistics of matched flood events over different regions**

1 Robustness of main results

1.1 Robustness to the alternative source of flood exposure data

The Global Flood Database (GFD)[1] is an alternative source of data to the Dartmouth Flood Observatory (DFO) database and also can be used to identify flood events. However, the GFD has a limited time span and total flood events, as it adopted satellite imageries to identify the flood events. Furthermore, GFD dataset does not provide comprehensive coverage of all areas affected by flood events. Instead, it focuses on specific regions with flood inundation. In addition, as the GFD dataset only captured about 30% of the global flood events, some participants may be misclassified as unexposed. To address the potential misclassification issue, we implemented additional screening measures in the matching results in this part of sensitivity analysis. Since the DFO covered nearly all flood events compared to the Global Flood Database GFD, any records that overlapped with a flood event in the DFO but not in the GFD were excluded from this part of analysis. By doing so, we ensured that the records included in this part of the analysis fell into one of the following categories: records with no exposure to floods at all, records exposed only to floods identified by the GFD, or records demonstrating the simultaneous recognition of flood events from both the GFD and DFO. Our aim in implementing this procedure was to minimize misclassification errors that could arise from solely considering unexposed data based on the GFD dataset.

In all, we use the GFD data to evolute the impact of exposure of flood inundation during pregnancy period, to test for the robustness of our main results. Table S1 listed the differences between identified cases and controls based on these two databases. As shown in Fig S1, although the matched cases were reduced based on GFD, the estimated risks of pregnancy loss are very similar.

Table S1: Characteristics of surveyed women who identified as experienced flood exposure one or more times during their previous gestation period based on two flood exposure databases.

Region	Total		Pregnancy loss		Successful delivery	
	DFO	GFD	DFO	GFD	DFO	GFD
Central American and Caribbean	3,335	3,112	679	625	2,642	2,112
South America	4,206	4,011	21,910	21,210	77,573	76,210
North Africa	235	213	6,592	5,321	17,468	15,967
Sub-Saharan Africa	9,212	8,903	8,734	7,431	21,950	20,210
Southern Africa	864	743	30,130	29,510	69,984	67,210
South Asia	16,653	15,810	1,435	1,112	4,792	4,015
West Asia	676	589	69,480	68,856	194,409	192,124

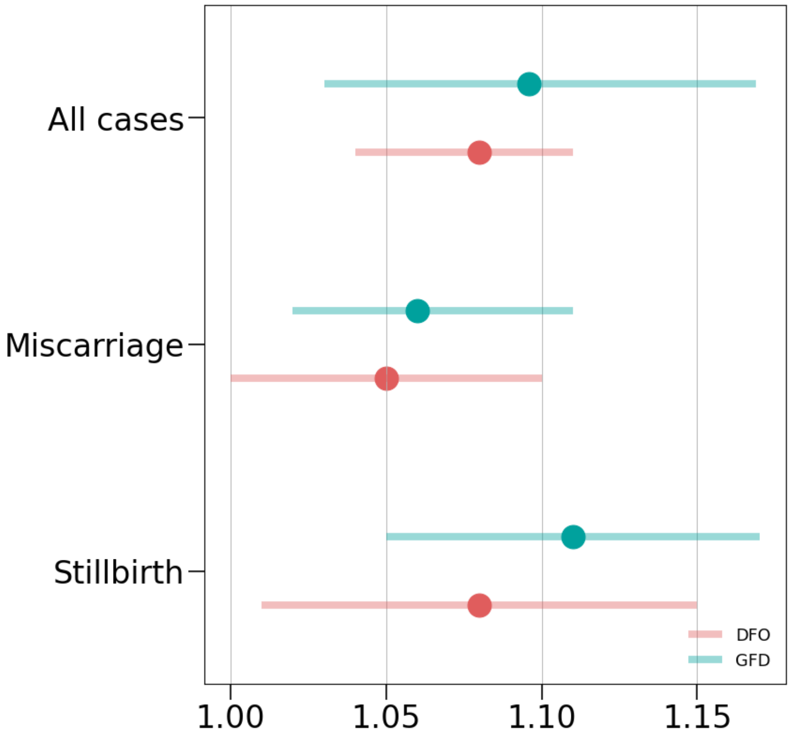

Fig S1: Change in the risk of pregnancy loss for women due to gestational flood exposure identified by two flood databases. The dots are point estimates of the odds ratio of pregnancy loss with gestational flood exposure. Error bars indicate 95% CIs.

1.2 Robustness to different controls

In the main model, we controlled maternal age in the delivery year and a categorical term for the year and month of conception, and gestational mean temperature and precipitation. Although this helps to improve the identification of the causal effect of flood events, such fixed effects restrict potentially important variation that occurs across time and space. Below, we test the sensitivity of our results to the inclusion of less restrictive control effects (Fig S2). All specifications imply that having flood exposure during the gestational period increases pregnancy loss risk, although the magnitude of the effect varies.

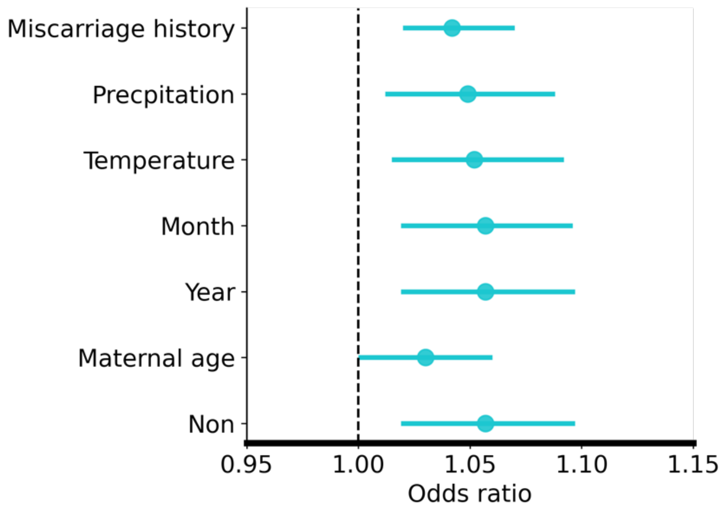

Fig S2 Estimation with different sets of covariates in the main model. The dots are point estimates and the error bars are corresponding 95% confidence intervals.

1.3 Robustness to leaving each region out individually

Our results from the main model may be driven by some regions with many cases. Below, we re-run our main model with cases from each main region individually. The dotted line represents the estimate reported in the main text. Results of all individual regions, as shown in Fig S3, are very close to the results from the main model.

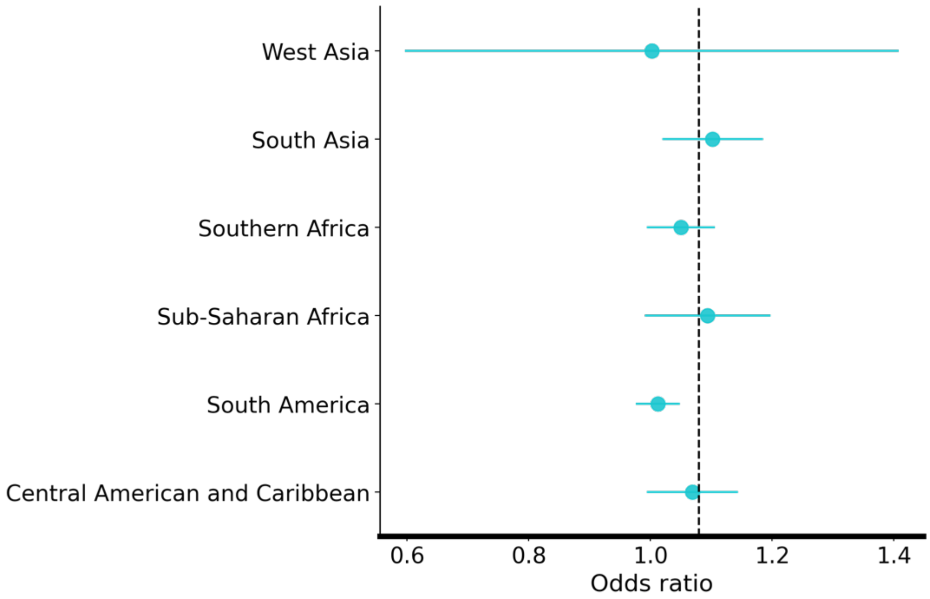

Fig S3 Estimation with cases from each of the main regions. The dashed line represents the mean odds ratio over all study regions. The dots are point estimates and the error bars are corresponding 95% confidence intervals.

1.4 Robustness to potential migration

Our primary findings are derived from the analysis of DHS surveys, which provide information on the location of clusters representing villages or neighborhoods where the mothers were interviewed. It is important to note that the house or location where the interview took place may not necessarily be the same as the place where the women resided during their previous gestational period, particularly in cases of forced migration due to floods. To address this potential bias, we decided to focus our analysis on a subset of women who reported having lived in their current house for at least 10 years. By applying this restriction, we excluded 72.73% of the total cases from our sample. As depicted in Figure S4, the main results obtained from this restricted sample remain consistent, albeit with wider error bars due to the reduced sample size.

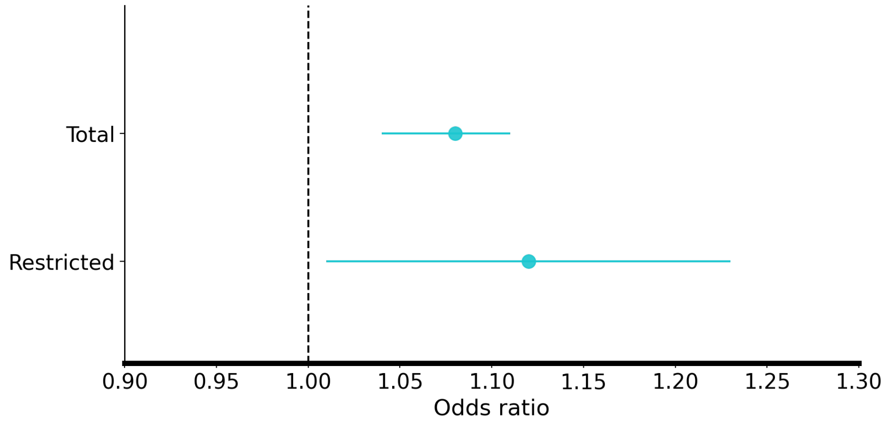

Fig S4: The main results and subsequently re-estimated the same parameters after excluding potential migrants from the sample. The dots are point estimates and the error bars are corresponding 95% confidence intervals.

2. Effects of different types of floods on pregnancy loss

Fig S5 depicts the excess pregnancy losses due to three main types of flood events. As mentioned in the main text, in addition to the total flood events, we estimated the excess pregnancy losses due to different sources of flood events, including heavy rains or monsoon rains, tropical cyclones or storms, and levee/dem break or release.

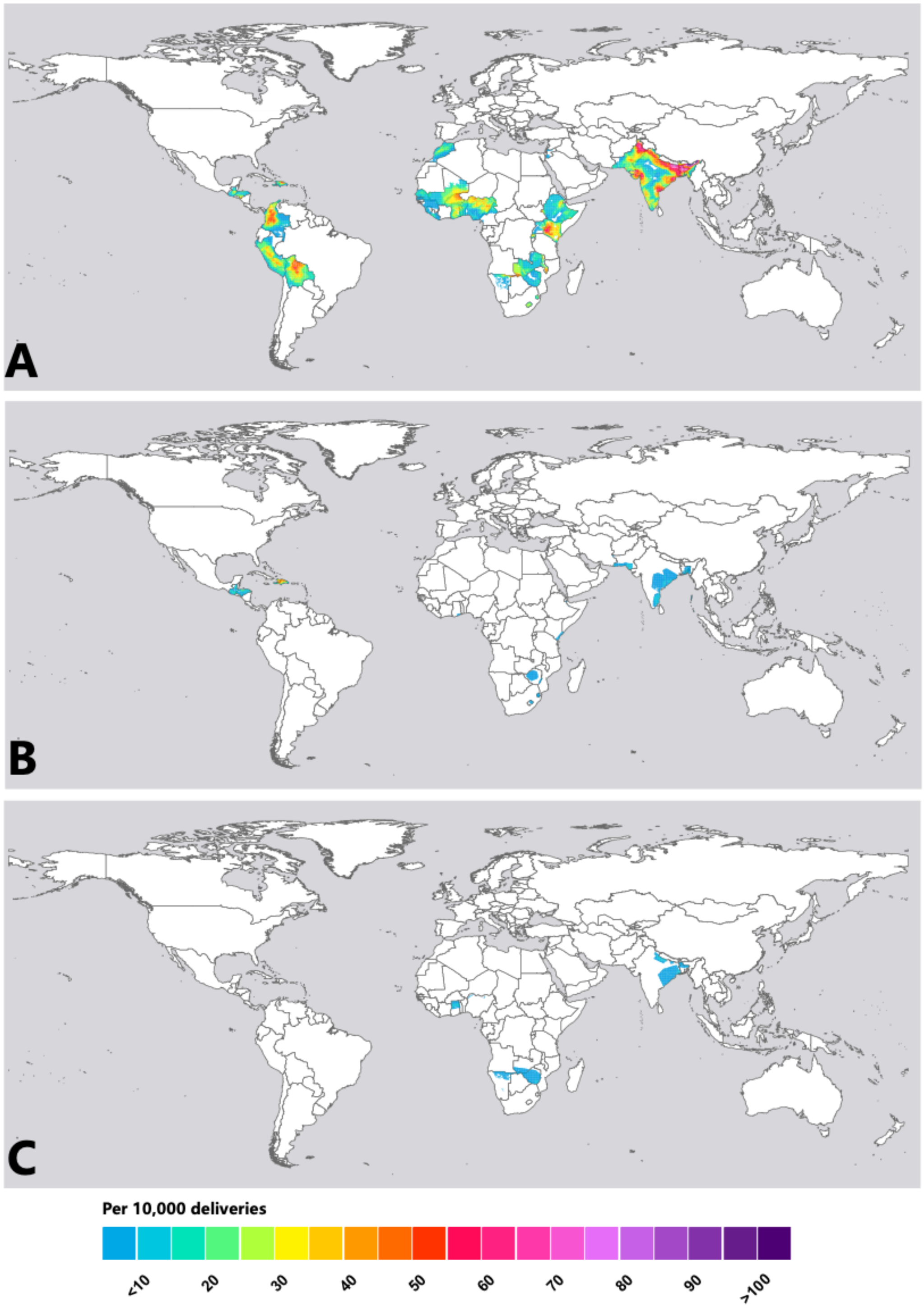

Fig S5: Average annual excess pregnancy losses (per 10,000 deliveries) due to three types of flood exposure (2010–2020). Spatial distribution of annual excess pregnancy losses at a spatial resolution of 10 km × 10 km due to floods sourced from heavy rains or monsoon rains (A), tropical cyclones or storms (B), and levee/dem break or release (C).

3. Extra data

4.1 Example illustrating our case-control design

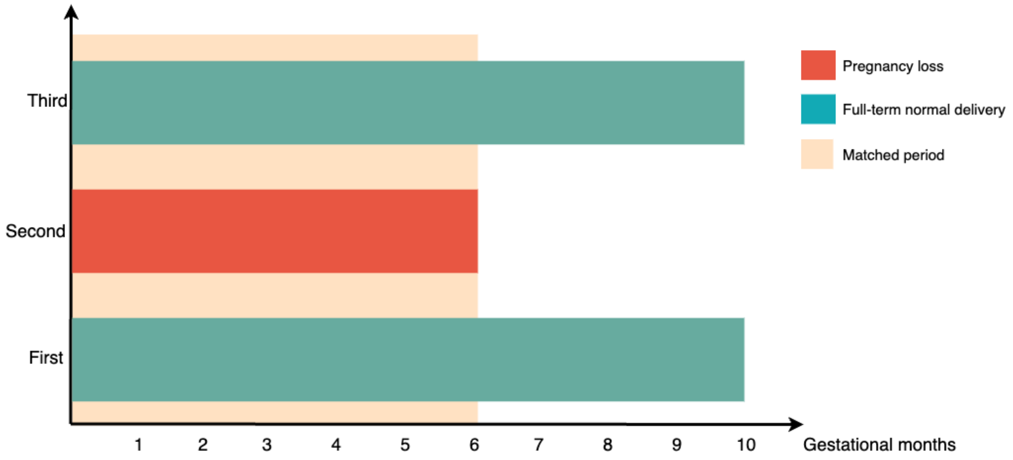

Fig S6: Example illustrating our case-control design. Bars represent the months of gestation for the cases.

4.1 Road map of data selection

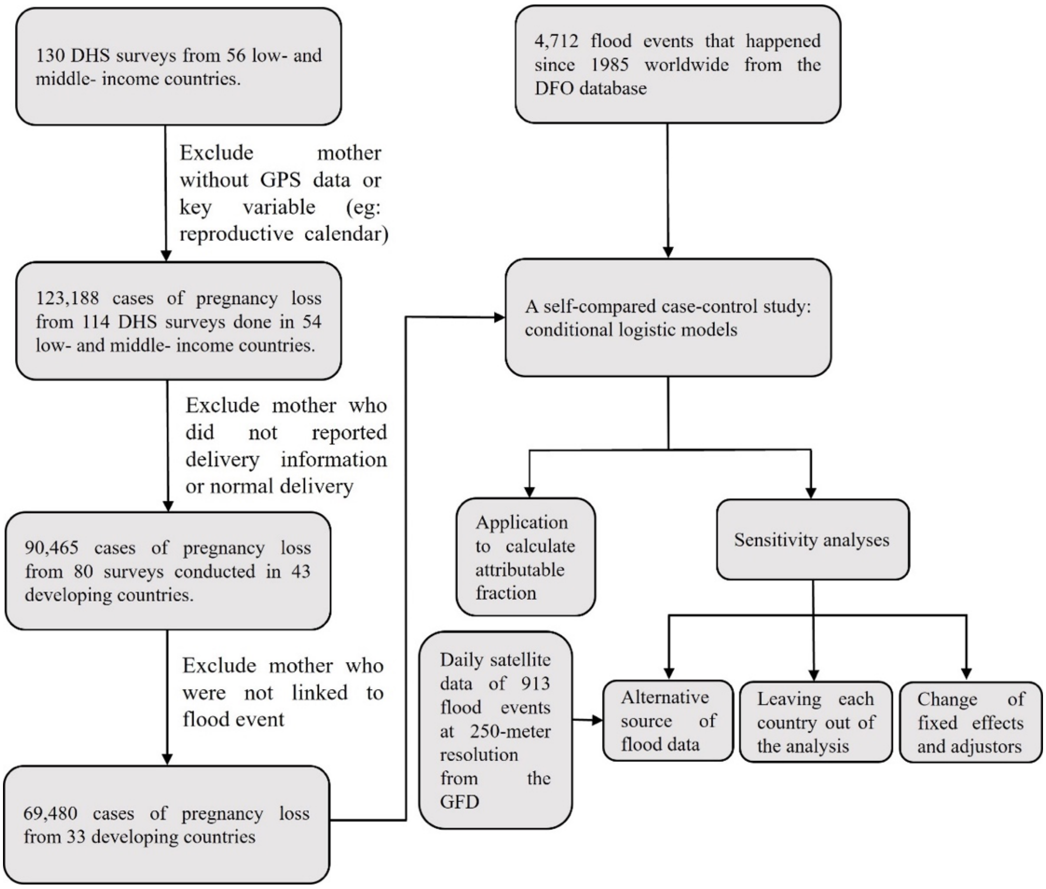

Fig S7: Road map of data collection and matching

4.2 DHS Surveys used in the main analysis

Table S2: Summary statistics for the surveyed women who have reported at least one successful pregnancy before or after the pregnancy loss, and have experienced flood exposure one or more times during their previous gestation period.

	Number of women	Successful delivery	Pregnancy loss
Bangladesh (2011)	522	3250	1396
Bangladesh (2014)	468	2487	1068
Benin (2011-12)	293	1295	415
Bolivia (2008)	663	3715	1365
Burkina Faso (2010)	401	4068	1186
Cameroon (2011)	523	6422	1791
Cameroon (2018)	355	3684	1112
Colombia (2010)	2021	6953	3177
Dominican Republic (2007)	998	4662	1947
Dominican Republic (2013)	394	2004	932
Eswatini (2006-07)	12	40	13
Ethiopia (2016)	361	2049	547
Ghana (2008)	220	1038	345
Ghana (2014)	339	2444	827
Guatemala (2014-15)	573	3071	1080
Guinea (2005)	206	1746	442
Guinea (2018)	276	1975	573
Haiti (2005-06)	223	1551	455
Haiti (2012)	144	597	251
Haiti (2016-17)	322	2148	759
Honduras (2011-12)	681	3435	1168
India (2015-16)	15055	60034	26600
Jordan (2017-18)	676	4792	1435
Kenya (2014)	466	2051	581
Lesotho (2009)	156	520	204
Lesotho (2014)	153	465	207
Liberia (2007)	235	2642	679
Malawi (2004)	319	1833	548
Malawi (2015-16)	514	2586	849
Mali (2006)	339	4152	955
Mali (2012-13)	223	1621	415
Mozambique (2011)	344	1853	629

Namibia (2006-07)	199	741	261
Nigeria (2003)	252	1930	467
Nigeria (2018)	1019	9271	2467
Pakistan (2006-07)	608	4213	1066
Peru (2004-06)	812	3999	1448
Peru (2009)	698	3284	1296
Rwanda (2014-15)	343	2061	653
Senegal (2005)	316	3802	950
Senegal (2012-13)	183	2312	640
Senegal (2014)	175	1858	500
Senegal (2016)	190	1891	525
Sierra Leone (2008)	201	1166	354
Togo (2013-14)	232	1534	480
Uganda (2016)	606	6553	1565
Zambia (2013-14)	427	2746	709
Zimbabwe (2005-06)	213	946	337
Zimbabwe (2010-11)	232	920	363

4.3 Statistics of matched flood events over different regions

Table S3 Summary statistics of matched flood events over different regions

	Total matched events	Mean overlapped days*
Central American and Caribbean	49	18
South America	27	37
North Africa	1	5
Sub-Saharan Africa	87	59
Southern Africa	2	6
South Asia	56	38
West Asia	2	2

* Overlapped days represents the overlapped period between flood affected period and gestation period of all matched records

References

1 Tellman, B. *et al.* Satellite imaging reveals increased proportion of population exposed to floods. *Nature* **596**, 80-86 (2021).

2 James, W. H. *et al.* Gridded birth and pregnancy datasets for Africa, Latin America and the Caribbean. *Scientific data* **5**, 1-11 (2018).

3 Tatem, A. J. *et al.* Mapping for maternal and newborn health: the distributions of women of childbearing age, pregnancies and births. *International journal of health geographics* **13**, 1-11 (2014).

4 Zhao, Q. *et al.* Global, regional, and national burden of mortality associated with non-optimal ambient temperatures from 2000 to 2019: a three-stage modelling study. *The Lancet Planetary Health* **5**, e415-e425 (2021).

5 Bearak, J., Popinchalk, A., Alkema, L. & Sedgh, G. Global, regional, and subregional trends in unintended pregnancy and its outcomes from 1990 to 2014: estimates from a Bayesian hierarchical model. *The Lancet Global Health* **6**, e380-e389 (2018).
