# Supplementary material for: Flood exposure and pregnancy loss in 33 developing countries
Source: Nat Commun. 2024 Jan 2;15:20. doi: 10.1038/s41467-023-44508-0 (PMC10761804; doi:10.1038/s41467-023-44508-0)
Supplement: Supplementary file 3 — Reporting Summary [file 41467_2023_44508_MOESM3_ESM.pdf]

nature portfolio

Corresponding author(s): Haidong Kan, Renjie Chen

Last updated by author(s): Nov 10, 2023

Reporting Summary

Nature Portfolio wishes to improve the reproducibility of the work that we publish. This form provides structure for consistency and transparency in reporting. For further information on Nature Portfolio policies, see our Editorial Policies and the Editorial Policy Checklist.

Statistics

For all statistical analyses, confirm that the following items are present in the figure legend, table legend, main text, or Methods section.

n/a	Confirmed	
☐	☒	The exact sample size (n) for each experimental group/condition, given as a discrete number and unit of measurement
☒	☐	A statement on whether measurements were taken from distinct samples or whether the same sample was measured repeatedly
☒	☐	The statistical test(s) used AND whether they are one- or two-sided<br>*Only common tests should be described solely by name; describe more complex techniques in the Methods section.*
☐	☒	A description of all covariates tested
☐	☒	A description of any assumptions or corrections, such as tests of normality and adjustment for multiple comparisons
☐	☒	A full description of the statistical parameters including central tendency (e.g. means) or other basic estimates (e.g. regression coefficient) AND variation (e.g. standard deviation) or associated estimates of uncertainty (e.g. confidence intervals)
☒	☐	For null hypothesis testing, the test statistic (e.g. F, t, r) with confidence intervals, effect sizes, degrees of freedom and P value noted<br>*Give P values as exact values whenever suitable.*
☒	☐	For Bayesian analysis, information on the choice of priors and Markov chain Monte Carlo settings
☐	☒	For hierarchical and complex designs, identification of the appropriate level for tests and full reporting of outcomes
☒	☐	Estimates of effect sizes (e.g. Cohen's d, Pearson's r), indicating how they were calculated

Our web collection on statistics for biologists contains articles on many of the points above.

Software and code

Policy information about availability of computer code

Data collection	No software was used to collect the date
Data analysis	All data analyses were completed using R statistical software (version 4.0.2) and Python (version 2.10.9). Statistical inference for the regression models was performed using the R package survival (version 3.3-1). The creation of Figure 1, 4a, and Supplementary Information (SI) Figures 5a to 5c was done using the Python package matplotlib (version 3.6.0).

For manuscripts utilizing custom algorithms or software that are central to the research but not yet described in published literature, software must be made available to editors and reviewers. We strongly encourage code deposition in a community repository (e.g. GitHub). See the Nature Portfolio guidelines for submitting code & software for further information.

Data

Policy information about availability of data

All manuscripts must include a data availability statement. This statement should provide the following information, where applicable:

- Accession codes, unique identifiers, or web links for publicly available datasets
- A description of any restrictions on data availability
- For clinical datasets or third party data, please ensure that the statement adheres to our policy

Survey data including pregnancy history records and socioeconomic data in this study are publicly available upon request from the Demographic and Health Surveys Program (https://dhsprogram.com/). Flood data are also publicly available from The Flood Observatory (https://floodobservatory.colorado.edu/). The number of pregnancies with a 1 km spatial resolution for each country was obtained from the WorldPop database (https://hub.worldpop.org/geodata/listing?id=19).

Research involving human participants, their data, or biological material

Policy information about studies with human participants or human data. See also policy information about sex, gender (identity/presentation), and sexual orientation and race, ethnicity and racism.

Reporting on sex and gender	No sex and gender information were involved in this study.
Reporting on race, ethnicity, or other socially relevant groupings	Our study obtained basic sociodemographic attributes of pregnant women from the DHS database, such as wealth, education, and residential area (urban or suburban), as well as fundamental living conditions including water sources and housing conditions. These attributes were recorded by field workers based on objective circumstances. We collected and used this data to analyze which specific social attributes of women are most affected by flooding.
Population characteristics	We included pregnancy records from a total of 35,181 eligible women from 33 developing countries out of 43 surveyed counties. Among these women, 69,480 pregnancy losses (i.e., cases) were matched to 194,409 successful deliveries (i.e., controls) (Table 1). On average, each case of pregnancy loss was matched with 4.52 controls, and the average wait time between cases and controls was 24.1 months. The average age at birth is 24
Recruitment	Our research is an observational study employing the comprehensive dataset gathered by field workers from the Demographic and Health Surveys (DHS). We have made use of every sample accessible in the DHS database.
Ethics oversight	NA. This study is based on publicly available population data, and we adhered to the DHS data usage guidelines in all our analyses. No further ethical approval is required.

Note that full information on the approval of the study protocol must also be provided in the manuscript.

Field-specific reporting

Please select the one below that is the best fit for your research. If you are not sure, read the appropriate sections before making your selection.

☒ Life sciences ☐ Behavioural & social sciences ☐ Ecological, evolutionary & environmental sciences

For a reference copy of the document with all sections, see nature.com/documents/nr-reporting-summary-flat.pdf

Life sciences study design

All studies must disclose on these points even when the disclosure is negative.

Sample size	We collected all the potential individual-level data of women with records of pregnancy loss from multiple Demographic and Health Surveys datasets worldwide. Then, we matched the flood database with the spatial information of each individual's living cluster. We identified a total of 35,181 eligible cases from 33 developing countries out of 43 surveyed countries. This included women who had experienced both fruitful pregnancies and pregnancy losses and had been exposed to floods during past pregnancies.
Data exclusions	We only included records from women who had experienced both fruitful pregnancies and pregnancy losses and had been exposed to floods during past pregnancies.
Replication	All attempts to repeat the experiment were successful.
Randomization	There was on experimental design or group allocation involved in this study
Blinding	There was on experimental design or group allocation involved in this study

Reporting for specific materials, systems and methods

We require information from authors about some types of materials, experimental systems and methods used in many studies. Here, indicate whether each material, system or method listed is relevant to your study. If you are not sure if a list item applies to your research, read the appropriate section before selecting a response.

Materials & experimental systems

n/a	Involved in the study
☒	☐ Antibodies
☒	☐ Eukaryotic cell lines
☒	☐ Palaeontology and archaeology
☒	☐ Animals and other organisms
☒	☐ Clinical data
☒	☐ Dual use research of concern
☒	☐ Plants

Methods

n/a	Involved in the study
☒	☐ ChIP-seq
☒	☐ Flow cytometry
☒	☐ MRI-based neuroimaging

Plants

Seed stocks	We hereby report that no specific seed stocks or other plant materials were utilized for the purpose of our study.
Novel plant genotypes	No novel plant genotypes were produced as part of our study.
Authentication	Since no seed stock use or novel genotype generation was involved in my study, there are no authentication procedures to describe, nor are there any experiments used to assess mutation effects or secondary effects such as off-target gene editing to report.
